# Unveiling the Value of Meta-Analysis in Disease Prevention and Control: A Comprehensive Review

**DOI:** 10.3390/medicina60101629

**Published:** 2024-10-05

**Authors:** Christos Ntais, Michael A. Talias

**Affiliations:** Healthcare Management Program, School of Economics & Management, Open University of Cyprus, Nicosia 2220, Cyprus; christos.ntais@st.ouc.ac.cy

**Keywords:** meta-analysis, disease prevention, disease control, clinical guidelines, health policy, big data, random-effects

## Abstract

Given the plethora of studies today that focus on the same topic, clinicians and other healthcare professionals increasingly rely on meta-analysis to aid in their evidence-based decision-making. This research method, which combines data from multiple studies to produce a single, more precise estimate of effect size, is invaluable for synthesizing evidence, resolving inconsistencies and guiding clinical practice and public health policies. Especially in disease prevention and control, meta-analysis has emerged as a critical tool. Meta-analysis is particularly valuable in assessing the effectiveness of preventive interventions such as vaccines, lifestyle modifications and screening programs. It provides robust evidence that supports the implementation of effective preventive measures and the discontinuation of ineffective or harmful ones. Furthermore, meta-analysis provides evidence to develop clinical practice guidelines, ensuring patients receive evidence-based treatments. In addition, public health policies aimed at disease prevention and control often rely on evidence from meta-analyses, which provide the data needed to justify and design large-scale public health initiatives. This comprehensive review delves into the role of meta-analysis in disease prevention and control, exploring its advantages, applications, challenges and overall impact on guiding clinical practice and public health policies. Through case studies and an examination of future directions, this paper underscores the pivotal role of meta-analysis in disease prevention and control.

## 1. Introduction

A meta-analysis is a quantitative approach that combines results from various studies addressing the same research question, and intends to derive a pooled effect size estimate that provides a more precise estimate of the intervention or exposure effect [1]. Meta-analysis seeks to overcome the shortcomings of individual studies, enhance the precision of results and offer a more reliable evidence base for decision-making by statistically combining different studies [1].

Meta-analysis is beneficial for medicine and health-related research as individual studies frequently provide different findings because of variations in their study design, sample size and population characteristics. This methodology is particularly valuable and has several applications in the context of disease prevention and control for a number of reasons, including synthesizing evidence, enhancing statistical power, identifying patterns and trends, evaluating interventions, addressing heterogeneity among individual studies, developing clinical guidelines [2] and guiding public health policies [3].

Despite the growing body of research on meta-analysis applications, there remains a significant gap in the literature regarding its specific value in enhancing disease prevention and control strategies. This review article aims to synthesize existing evidence and highlight specific case studies where meta-analysis has informed effective public health interventions and has influenced decision-making in disease prevention and control. Understanding the practical implications of meta-analyses in disease prevention and control can illuminate how research findings can be applied in real-world settings. By integrating findings from different diseases and interventions, this review offers a holistic view of meta-analysis’s effectiveness in the context of disease prevention and control.

## 2. Methods

The method used was the narrative literature review. Articles for this review were selected through a comprehensive literature search. We utilized multiple databases, including PubMed, the Cochrane Library and Scopus, and employed a combination of keywords such as “meta-analysis”, “disease prevention”, “disease control”, “clinical guidelines”, “health policy” and “public health”. The inclusion criteria focused on peer-reviewed articles published up to August 2024 that specifically examined the application of meta-analysis in public health contexts, particularly in relation to infectious and chronic diseases with significant societal, health and economic burden. Studies were prioritized based on their relevance to disease prevention and control strategies. Reference lists of identified articles were screened for additional relevant studies, ensuring a thorough exploration of the existing literature. This approach aimed to provide a well-rounded perspective on how meta-analysis can enhance decision-making and effectiveness in disease prevention and control efforts.

## 3. Meta-Analysis vs. Systematic Review

While meta-analyses and systematic reviews are research methods used to synthesize previous studies, their purpose and approach differ [4]. A systematic review employs a structured methodology to provide an exhaustive summary of all pertinent research on a topic and offers a qualitative synthesis of the literature. On the other hand, a meta-analysis is a statistical method that generates a quantitative estimate of the effect size by combining data from several studies that address the same research question. Table 1 outlines the main characteristics of systematic review and meta-analysis.

## 4. Meta-Analysis Step-by-Step Conduct

### 4.1. Formulating the Research Question

The first step in conducting a meta-analysis is formulating a clear, focused research question [5]. This involves defining the participants, interventions, comparisons, outcomes and study designs (PICOS). A well-defined research question guides the subsequent stages of the meta-analysis process.

### 4.2. Literature Search and Study Selection

A comprehensive literature search is conducted to identify relevant studies. The ideal number of databases to search for studies included in a meta-analysis typically depends on the research question, the field of study and the availability of relevant literature. Generally, searching three to five key databases is a common practice to ensure a comprehensive search of the literature. For public health topics, databases like PubMed, the Cochrane Library and Embase are often prioritized due to their extensive coverage of clinical trials [6]. The search strategy should include keywords and Boolean operators to capture all relevant studies [7]. Inclusion and exclusion criteria are established to select studies that meet the predefined eligibility criteria.

### 4.3. Data Extraction

Data extraction involves systematically collecting relevant information from each individual study. This includes details on study design, population characteristics, interventions, outcomes and results. Standardized data extraction forms are often used to ensure consistency and accuracy [5]. Employing multiple extractors allows for a more thorough and comprehensive assessment of the included studies as each extractor may bring different perspectives and expertise. This collaborative approach can strengthen the transparency and reproducibility of the research, as documented discrepancies and the consensus process can be reported. Overall, utilizing more than one data extractor is a practice that contributes significantly to the integrity and credibility of meta-analytic findings [8].

### 4.4. Assessing Study Quality

The quality of included studies is assessed using established tools, such as the Cochrane Risk of Bias Tool for randomized controlled trials [9] or the Newcastle-Ottawa Scale for observational studies [10]. Assessing study quality is crucial for interpreting the findings of the meta-analysis and identifying potential sources of bias. Having more than one researcher involved in checking the quality of the studies included in a meta-analysis is essential for ensuring the validity and reliability of the findings. This collaborative approach helps to mitigate individual biases and reduces the risk of oversight that can occur when a single researcher assesses study quality. By employing multiple researchers to check the quality of the studies, discrepancies in quality assessments can be identified and discussed, leading to more rigorous evaluation criteria and enhanced consensus on the quality of the included studies [11].

### 4.5. Statistical Analysis

Statistical analysis in meta-analysis involves combining the results of individual studies to produce a pooled estimate. The choice of statistical model depends on the degree of heterogeneity among the studies. Fixed-effects models assume that all studies estimate the same underlying effect, while random-effects models account for variability among studies [12,13]. In general, when combining results from different studies in a meta-analysis, several key considerations are essential to ensure the validity and reliability of the findings, including heterogeneity, study quality, the standardization of outcome measures, publication bias and the variability in settings, populations and interventions across studies.

### 4.6. Heterogeneity Assessment

Heterogeneity refers to the variability among study results. Heterogeneity in meta-analysis is typically classified as statistical heterogeneity and clinical heterogeneity. Statistical heterogeneity refers to the variation in study results that cannot be attributed to chance alone. It is often assessed using statistical methods, such as the Cochran’s Q test and the I^2^ statistic [14]. Clinical heterogeneity refers to differences in study characteristics that may affect the outcomes, such as population differences, intervention differences and outcome measures [15]. For instance, in a meta-analysis of drug trials, heterogeneity might occur if studies include patients with varying severities of their condition or use different doses of the medication.

Both types of heterogeneity can influence the interpretation of meta-analytic results. Researchers may explore sources of heterogeneity through subgroup analyses or sensitivity analyses, and when substantial heterogeneity is present, they might choose to use random-effects models instead of fixed-effect models to account for the variability in study results [15]. Understanding and addressing heterogeneity is crucial for drawing reliable conclusions from meta-analyses.

### 4.7. Publication Bias Assessment

**Figure 1:**
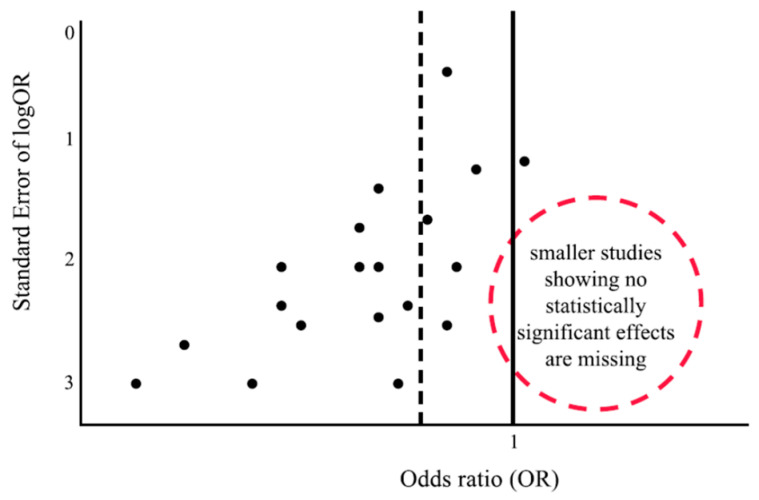
Hypothetical asymmetrical funnel plot in the presence of publication bias (the dashed line is the pooled effect; the solid line is the null effect).

Publication bias in meta-analysis refers to the tendency for studies with statistically significant or favorable results to have a higher likelihood of being published than those with null or negative findings [16]. This bias skews the data pool as unpublished studies are usually excluded, leading to an overestimation of the effect sizes in the meta-analysis. It can distort conclusions and reduce the validity of the findings, potentially misleading researchers, policymakers and clinicians. Techniques such as funnel plots [17] and statistical tests [18,19], such as Egger’s test, are used to detect publication bias. A hypothetical example of an asymmetrical funnel plot in the presence of publication bias is illustrated in Figure 1.

To adjust for publication bias in meta-analysis, the “trim-and-fill” method can be used [20]. Firstly, the method identifies asymmetry in a funnel plot, which is often a sign of publication bias. Studies that cause this asymmetry, typically those with small sample sizes and extreme effect sizes, are “trimmed” from the analysis to create a more symmetrical plot. Then, the method “fills” in the missing studies by imputing hypothetical data points, assuming that some small or non-significant studies are missing from the analysis due to publication bias. These imputed studies are added to balance the funnel plot and provide an adjusted estimate of the overall effect size that accounts for potential bias [21]. By applying the trim-and-fill method, researchers can obtain a more accurate and less biased estimate of the true effect size in a meta-analysis.

### 4.8. Interpretation and Reporting

The final step involves interpreting the results and reporting the findings transparently and comprehensively. The Preferred Reporting Items for Systematic Reviews and Meta-Analyses (PRISMA) guidelines provide a standardized framework for reporting meta-analyses [22]. Key elements include the study selection process, data extraction, quality assessment, statistical methods and main findings.

## 5. Advantages of Meta-Analysis

### 5.1. Enhanced Statistical Power

Individual studies often need a larger sample size to detect small but clinically significant effects. By combining data from several studies, meta-analysis enhances statistical power and the capacity to detect true associations or intervention effects. This increased power is essential for detecting modest benefits of preventive measures or subtle risk factors that may not be identified in smaller studies. For example, meta-analyses of studies on the efficacy of influenza vaccines have demonstrated significant benefits that individual studies with limited sample sizes could not reliably detect [23].

### 5.2. Comprehensive Evidence Synthesis

Meta-analysis provides a more comprehensive and representative view of the existing evidence by combining results from various studies. Understanding how interventions perform across multiple populations, settings and contexts is particularly important for disease prevention and control, which is where this thorough synthesis comes in. By integrating findings from diverse studies, meta-analysis contributes to the formation of a holistic understanding of disease dynamics and intervention outcomes. For instance, meta-analyses of dietary interventions to prevent cardiovascular disease integrate data from diverse populations, leading to more generalized and applicable recommendations [24].

### 5.3. Identification of Consistent Patterns

Identifying recurring patterns and trends across multiple studies is one of the strengths of meta-analysis. This can be critical for understanding the epidemiology of diseases, determining common risk factors and assessing the effectiveness of preventive strategies. Meta-analyses have been pivotal in the identification of consistent links between lifestyle variables (such as diet, physical activity and smoking) and chronic diseases like heart disease and cancer [25,26].

### 5.4. Resolving Conflicting Findings

Conflicting or contradictory findings from individual studies can induce uncertainty and impede the implementation of effective disease prevention and control measures. By offering a pooled estimate that reflects the effect’s overall direction and magnitude, meta-analysis helps resolve these conflicts. This is particularly useful in controversial research areas with disparate findings. For example, meta-analyses have clarified the benefits of statin use in the primary prevention of cardiovascular disease, despite individual studies showing inconsistent results [27].

## 6. Meta-Analysis Case Studies in Disease Prevention and Control

Specific case studies are presented below that illustrate how healthcare providers and public health policymakers have successfully translated meta-analytic findings into practical interventions that enhance disease prevention and control efforts.

### 6.1. Vaccination Programs

#### 6.1.1. Influenza Vaccination

Influenza poses a significant public health threat, particularly among vulnerable populations such as the elderly and individuals with chronic conditions. Meta-analyses have provided robust evidence on the effectiveness of influenza vaccines in preventing flu-related morbidity and mortality [23]. For instance, a meta-analysis confirmed the efficacy of influenza vaccines in reducing the incidence of laboratory-confirmed influenza [23]. This evidence supports annual vaccination recommendations and informs public health strategies to mitigate the impact of seasonal flu.

#### 6.1.2. Human Papillomavirus (HPV) Vaccination

Meta-analyses also play a crucial role in evaluating the efficacy of vaccines and addressing the public’s concerns about potential adverse effects. HPV is a leading cause of cervical cancer. Meta-analyses have demonstrated the safety and effectiveness of HPV vaccines in preventing cervical cancer [28]. More specifically, a meta-analysis demonstrated that HPV vaccination significantly reduces the incidence of cervical precancerous lesions and HPV infection [28]. The meta-analysis included data from multiple randomized controlled trials, providing robust evidence on the protective effects of HPV vaccines. These findings have informed global vaccination programs and have led to widespread adoption of HPV vaccines in national immunization programs.

### 6.2. Screening Programs

#### 6.2.1. Mammography Screening

Breast cancer is a significant cause of morbidity and mortality among women. Mammography screening is an essential tool for early detection and prevention of breast cancer. Meta-analyses have been instrumental in evaluating the benefits and risks of mammography screening [29]. A meta-analysis assessed the effectiveness of mammography screening in reducing breast cancer mortality, concluding that regular mammography screening significantly reduces breast cancer mortality, particularly in women aged 50–69 years [29]. These findings have informed clinical guidelines that recommend routine mammography for the early detection of breast cancer.

#### 6.2.2. Colorectal Cancer Screening

Colorectal cancer screening is essential for the early detection and prevention of colorectal cancer. Meta-analyses have assessed the effectiveness of various screening methods, such as colonoscopy, flexible sigmoidoscopy, computed tomographic colonography (CTC), fecal occult blood testing and fecal immunochemical testing [30]. A meta-analysis demonstrated that colonoscopy, flexible sigmoidoscopy, CTC and stool tests have differing levels of evidence to support their use, including their ability to detect cancer and precursor lesions and their risk of serious adverse events in average-risk adults [30]. These findings have guided screening recommendations and have increased the adoption of effective screening programs.

### 6.3. Lifestyle Interventions

#### 6.3.1. Smoking Cessation

Smoking is a leading cause of preventable morbidity and mortality worldwide. Meta-analyses have evaluated the effectiveness of various smoking cessation interventions, including pharmacological treatments, behavioral therapies and policy measures such as smoking bans and tobacco taxes [31,32]. For example, a meta-analysis evaluated the effectiveness of pharmacological interventions for smoking cessation [31]. The meta-analysis included data from multiple randomized controlled trials and found that nicotine replacement therapy, bupropion and varenicline significantly increase smoking cessation rates compared to placebo. Another meta-analysis found that comprehensive smoke-free laws and increased tobacco taxes significantly reduce smoking prevalence and cigarette consumption [32]. These findings have informed public health initiatives aimed at reducing smoking prevalence and its associated health risks.

#### 6.3.2. Physical Activity

Regular physical activity prevents chronic diseases such as cardiovascular disease, diabetes and obesity. Meta-analyses have provided evidence on the health benefits of physical activity [26]. A meta-analysis concluded that physical activity benefits cardiovascular health by reducing the overall risk of incident coronary heart disease and stroke [26]. These findings support public health initiatives to increase physical activity levels among the general population.

### 6.4. Pharmacological Interventions

#### 6.4.1. Statin Therapy

Cardiovascular disease (CVD) is a leading cause of morbidity and mortality worldwide. Statins are widely used to lower cholesterol levels and reduce the risk of cardiovascular incidents. Meta-analyses have provided robust evidence supporting the efficacy of statins in both the primary and secondary prevention of cardiovascular disease [27]. For instance, a meta-analysis evaluated the effects of statin therapy on cardiovascular outcomes [27]. The meta-analysis included data from 26 randomized trials and found that statin therapy reduces the risk of major cardiovascular events by approximately 20% for each 1 mmol/L reduction in LDL cholesterol. These findings have influenced clinical guidelines worldwide that recommend statin therapy for individuals at high risk of cardiovascular disease.

#### 6.4.2. Antihypertensive Medications

Hypertension is a significant risk factor for cardiovascular disease. Meta-analyses have evaluated the effectiveness of various blood pressure-lowering medications in reducing cardiovascular risk [33]. A meta-analysis demonstrated that antihypertensive drugs are effective in reducing the risk of stroke and heart disease across different patient populations [33]. Additionally, meta-analyses have compared the effectiveness of various classes of antihypertensive drugs [34]. A meta-analysis concluded that all major classes of antihypertensive drugs are similarly effective in preventing cardiovascular events, providing flexibility in treatment options [34]. These findings have informed guidelines recommending blood pressure control to prevent cardiovascular incidents.

### 6.5. Dietary Interventions

#### 6.5.1. Mediterranean Diet

The Mediterranean diet, characterized by the high consumption of fruits, vegetables, whole grains and healthy fats, has numerous health benefits. Meta-analyses have provided evidence on the protective effects of the Mediterranean diet against various chronic diseases [25,35]. For example, a meta-analysis found that adherence to the Mediterranean diet significantly reduces the risk of cardiovascular disease, cancer and overall mortality [35]. Another meta-analysis found that higher fruit and vegetable intake is associated with a lower risk of cardiovascular disease, cancer and all-cause mortality [25]. These findings have promoted the adoption of the Mediterranean diet as a dietary pattern for disease prevention and health promotion [36].

#### 6.5.2. Low-Carbohydrate Diets

Low-carbohydrate diets have gained popularity for weight management and metabolic health. Meta-analyses have evaluated the effects of low-carbohydrate diets on weight loss and cardiovascular risk factors [37]. A meta-analysis found that low-carbohydrate diets are effective for short-term weight loss and improve cardiovascular risk factors, such as HDL cholesterol and triglyceride levels [37]. These findings support the inclusion of low-carbohydrate diets in clinical practice as an option for weight management and metabolic health [38].

## 7. Meta-Analysis Case Studies with Inconclusive or Conflicting Results

Despite their indisputable value in the field of disease prevention and control, there are several instances where meta-analyses have yielded inconclusive or conflicting results, often due to variations in the study quality, heterogeneity in the methods or the presence of biases. A few notable examples are presented below.

### 7.1. Vitamin D Supplementation and Cancer Prevention

Multiple meta-analyses have examined the role of vitamin D supplementation in cancer prevention [39,40]. Some analyses suggest a protective effect while others find no significant association. Indicatively, a meta-analysis showed that vitamin D does not reduce cancer incidence regardless of whether the supplement is given daily or in a large bolus while, among trials testing daily supplementation, a significant benefit is observed among normal-weight individuals [39]. Another meta-analysis that included data from 50,623 individuals found that vitamin D does not significantly reduce cancer occurrence but that it significantly decreases cancer mortality by 12%; however, this finding could be due to random errors [40].

### 7.2. Aspirin for Cardiovascular Disease Prevention

Aspirin has been widely studied for its role in preventing CVD [41,42]. A meta-analysis has shown that while low-dose aspirin reduces the risk of major adverse cardiovascular events (MACE) in high-risk populations, its benefits in low-risk individuals are less clear, particularly given the increased risk of bleeding events [41]. Another meta-analysis that included data from 173,810 individuals found that aspirin significantly reduces the risk of MACE by 11% but increases the risk of major bleeding, intracranial hemorrhage and gastrointestinal bleeding, leading to inconclusive recommendations for its use in primary CVD prevention [42].

### 7.3. Omega-3 Fatty Acids and Cardiovascular Health

Meta-analyses on the role of omega-3 fatty acid supplements in cardiovascular health have yielded inconsistent results [43,44]. Some show a reduction in MACE, while others find no significant protective effects. Indicatively, a meta-analysis has shown that omega-3 fatty acids may reduce the risk of MACE, myocardial infarction and cardiovascular death, but the potential risk of atrial fibrillation and bleeding cannot be ignored. In addition, omega-3 fatty acids may increase the risk of stroke in patients with myocardial infarction [43]. A Cochrane review concluded that omega-3 supplements have little or no effect on cardiovascular mortality or events [44].

## 8. Challenges and Limitations of Meta-Analysis

### 8.1. Heterogeneity

Heterogeneity, or variability among studies, is a common challenge in meta-analysis. Differences in study design, populations, interventions and outcomes can contribute to heterogeneity, complicating the interpretation of results [45]. Statistical methods, such as meta-regression and subgroup analyses, are used to address heterogeneity. For example, in meta-analyses on cancer treatments, researchers can perform subgroup analyses based on tumor stage, age or other clinical variables to clarify which patients benefit most from a specific treatment. Meta-regression goes further by statistically testing the relationship between study characteristics (e.g., dose, duration) and outcomes, allowing for a deeper understanding of heterogeneity.

### 8.2. Publication Bias

Publication bias occurs when studies with significant findings are more likely to be published than those with null results [16]. This bias can skew the results of a meta-analysis and lead to the overestimation of treatment effects [46]. Performing comprehensive literature searches and including unpublished data or data from clinical trial registries can mitigate its impact.

### 8.3. Quality of Included Studies

The quality of studies included in a meta-analysis affects the reliability and validity of the findings. Low-quality studies with methodological flaws can introduce bias and affect the overall conclusions. Therefore, it is essential to use rigorous criteria for study selection and to conduct quality assessments of included studies using standardized tools, such as the Cochrane Risk of Bias Tool [9]. Excluding low-quality studies or conducting sensitivity analyses can also help mitigate the impact of study quality on the overall findings.

### 8.4. Data Availability and Reporting

Incomplete or inconsistent reporting of study data can pose challenges for data extraction and analysis in meta-analysis. Limited access to raw data, selective reporting of outcomes and inadequate reporting of study details can limit the ability to conduct a comprehensive meta-analysis. Promoting transparency and adherence to reporting guidelines can improve the quality and completeness of reporting in primary studies.

### 8.5. Evolving Evidence Base

The evidence base in healthcare is continually evolving, with new studies being published regularly. This can affect the relevance and currency of meta-analysis findings. Meta-analyses must be periodically updated to incorporate the latest evidence and ensure that conclusions remain current and relevant. Living meta-analyses that are continuously updated as new evidence becomes available represent an innovative approach to addressing this challenge [47]. Practical examples of living meta-analyses can be seen in the response to the COVID-19 pandemic, where treatments, vaccines and prevention strategies have been under constant evaluation. For instance, COVID-NMA, an international research initiative supported by the World Health Organization and the Cochrane Collaboration, implemented living meta-analyses to continuously assess the effectiveness of interventions for COVID-19. As new trials were published, their data were incorporated to provide the most current recommendations for healthcare providers and policymakers.

## 9. Meta-Analysis in a “Big Data” World

While meta-analysis is widely recognized for assimilating data from larger populations than individual studies, other approaches, such as big data, can also yield conclusions from huge sample sizes. Big data is the term used to describe datasets that are too large or too specific in type to be managed and processed by standard data processing software [48]. In the field of medicine in particular, big data allows the analysis of information from thousands of patients to find patterns or associations between variables gathered from various datasets. Big data analysis may drive advancements in personalized medicine by evaluating risks, predicting outcomes and preventing undesired variability [49]. Attractive as it may be, the validity, accuracy and value of the data obtained remain questionable [49,50].

The integration of big data in meta-analyses introduces several ethical challenges, particularly regarding data privacy and the quality of real-world evidence (RWE) [51]. In terms of privacy, large datasets from sources like electronic health records (EHRs) and wearable devices can potentially expose sensitive patient information, even when anonymized, as re-identification risks increase when data from multiple sources are combined. This raises concerns about informed consent, as patients may not be fully aware their data are being used in secondary research. Additionally, the quality of RWE often varies. It can be incomplete, inconsistently collected or biased, particularly if certain populations (e.g., low-income or minority groups) are underrepresented. These data quality issues can lead to misleading conclusions in meta-analyses and compromise the validity of the findings.

In the context of disease prevention and control, the comparison between meta-analysis and big data highlights distinct yet complementary approaches to evidence synthesis and decision-making. Meta-analysis focuses on systematically combining results from multiple studies to generate precise estimates of intervention effectiveness, allowing for rigorous evaluations of established research. This method is particularly valuable for assessing the efficacy of specific public health interventions based on curated datasets. In contrast, big data encompasses vast and complex datasets from diverse sources, such as EHRs, social media and wearable devices, enabling real-time analysis and insights into population health trends. While meta-analysis offers structured and systematic evaluations, big data allows for the exploration of emerging patterns and associations that may not be captured in traditional research. Together, they provide a more holistic view: meta-analysis can validate findings derived from big data analyses, while big data can inform and refine the questions addressed in future meta-analyses, ultimately enhancing strategies for disease prevention and control.

## 10. Discussion

Clinical practice guidelines are essential for standardizing care and ensuring patients receive evidence-based treatments. Meta-analyses provide the robust evidence needed to develop these guidelines. For example, guidelines for managing diabetes often rely on meta-analyses that evaluate the efficacy of different treatments in achieving glycemic control [52]. A meta-analysis compared the efficacy of various glucose-lowering drugs, providing evidence that informs treatment decisions in clinical practice [52]. Furthermore, meta-analyses have evaluated the benefits of lifestyle interventions in diabetes management [53]. A meta-analysis demonstrated that structured exercise interventions significantly improve glycemic control in patients with type 2 diabetes, supporting the inclusion of physical activity in diabetes management guidelines [53].

Meta-analysis informs health policy decisions by providing comprehensive evidence on the efficacy and safety of interventions. Policymakers rely on meta-analyses to allocate resources, prioritize public health initiatives and design and implement evidence-based, large-scale public health interventions. For example, obesity is a major public health concern linked to various chronic diseases, and public health policies aimed at obesity prevention rely on evidence from meta-analyses that have examined the impact of dietary and physical activity interventions on weight management [54,55]. A meta-analysis evaluated the impact of school-based interventions in preventing childhood obesity, finding that these programs can reduce the prevalence of obesity among children [54]. Another meta-analysis found that all major dietary programs result in modest weight loss with no significant differences between them, suggesting that adherence to a diet is more important than the specific diet itself [55]. These findings inform policies and programs aimed at curbing the obesity epidemic.

## 11. Conclusions

By synthesizing data from multiple studies, meta-analysis plays a pivotal role in disease prevention and control. Individual studies often yield varying results due to differences in their study design, sample size and population characteristics. Meta-analysis enhances the statistical power to detect effects, offering robust and comprehensive insights into the efficacy of interventions. Moreover, meta-analyses provide high-quality evidence to develop clinical practice guidelines and inform public health policies. This approach enables health authorities to identify best practices, assess risks and design large-scale public health interventions. Meta-analyses can improve patient outcomes and advance public health by ensuring these interventions are based on the best available data. As global health challenges continue to evolve, the importance of meta-analysis in guiding effective disease prevention and control strategies remains indispensable.

## 12. Future Directions

The field of meta-analysis continues to evolve, with advancements in statistical methods, integration of RWE and innovative approaches to evidence synthesis such as living meta-analyses and umbrella reviews.

### 12.1. Advanced Statistical Methods

The development of advanced statistical techniques, such as network meta-analysis (NMA) [56] and individual participant data (IPD) meta-analysis [57], allows for more sophisticated analyses and comprehensive evidence synthesis. NMA allows for the comparison of multiple interventions by incorporating both direct comparisons (e.g., from head-to-head trials) and indirect comparisons (where treatments are linked through a common comparator) into a unified analysis. This method is especially useful in evaluating the relative effectiveness of different treatments, even when direct evidence is limited or unavailable. IPD meta-analysis, on the other hand, involves obtaining and re-analyzing raw data from each participant across multiple studies. This allows for a more detailed and precise analysis, enabling subgroup analyses and the exploration of patient-level factors that influence outcomes, which aggregate data meta-analyses may not achieve. Both approaches enhance the robustness of evidence-based decisions in healthcare, with NMA focusing on treatment comparisons and IPD meta-analysis offering deeper insights into individual variability in treatment effects.

### 12.2. Integration of Real-World Evidence

Integrating RWE from EHRs, registries and other data sources into meta-analyses can enhance the applicability and relevance of findings [58]. RWE provides valuable insights into the effectiveness and safety of interventions in routine clinical practice, complementing evidence from randomized controlled trials.

### 12.3. Living Meta-Analyses

Living meta-analyses represent an innovative evidence synthesis approach that involve continuously updating pooled estimates as new evidence becomes available [47]. This means that the effect estimates can change as the evidence base grows. This dynamic approach ensures that findings remain current and relevant, and support timely decision-making in clinical practice and public health policy. For instance, living meta-analyses were widely used during the COVID-19 pandemic to keep up with the rapidly emerging research on treatments, vaccines and preventive measures [59]. Technological advances like machine learning can aid in efficiently updating living meta-analyses by automating and streamlining various stages of the process [60]. One of the primary benefits of machine learning is its ability to rapidly identify relevant studies and extract key information from large volumes of literature. Machine learning algorithms can be trained to recognize patterns and categorize studies based on predefined criteria, significantly reducing the time researchers spend on manual searches and data extraction.

### 12.4. Umbrella Reviews

Traditionally, the highest level of evidence synthesis has been represented by systematic reviews and meta-analyses [61]. But over the past few decades, the quantity of systematic reviews and meta-analyses has steadily grown to the point where some sectors are overrun [62]. Moreover, some meta-analyses overlap but come to different conclusions [63]. Umbrella reviews are a type of evidence synthesis that have emerged in recent years to provide a broad overview of the extensive evidence on a particular topic [64]. Umbrella reviews summarize and stratify the quality or strength of the evidence from earlier systematic reviews or meta-analyses performed on a specific topic. Authors of umbrella reviews frequently employ several strategies to address discrepancies in overlapping meta-analyses. For instance, they may present the findings of the meta-analysis with the largest number of studies to reduce biases brought on by the non-identification of relevant studies [65]. Alternatively, they may present the meta-analysis results demonstrating the highest methodological quality [66]. Overall, umbrella reviews point readers to the most up-to-date evidence by summarizing extensive information in a single publication.

## Figures and Tables

**Table 1:** Main Characteristics of Meta-Analysis and Systematic Review.

	Meta-Analysis	Systematic Review
Purpose	To derive more precise and reliable conclusions by pooling results from multiple studies addressing the same question	To provide a reliable, objective and comprehensive summary of existing literature on a specific topic
Methodology	Follows a systematic search and selection process of relevant studies	Follows a systematic search and selection process of relevant studies
Data synthesis	Quantitatively synthesizes data using statistical techniques	Qualitatively summarizes findings from included studies
Inclusion criteria	Clearly predefines inclusion and exclusion criteria	Clearly predefines inclusion and exclusion criteria
Quality assessment	Uses quality assessment tools, e.g., Cochrane Risk of Bias Tool, Newcastle-Ottawa Scale	Critically appraises included studies
Analysis method	Quantitatively analyzes primary study data, often includes meta-regression and sensitivity analysis	Qualitatively analyzes and synthesizes study findings
Outcome	Provides a pooled estimate of effect size	Summarizes evidence and identifies research gaps
Impact	Guides clinical practice, informs health policy decisions	Guides clinical practice, informs health policy decisions
Advantages	Enhances statistical power, provides a comprehensive evidence synthesis, identifies consistent patterns, resolves uncertainty and conflicts	Provides an exhaustive summary of evidence on a specific topic
Limitations	Limited by availability and quality of primary study data	Limited by subjectivity in synthesis, availability and quality of included studies

Source: Authors’ own work.

## Data Availability

Not applicable.
